# The structural protein VP3 of enterovirus D68 interacts with MAVS to inhibit the NF-κB signaling pathway

**DOI:** 10.1128/jvi.00163-25

**Published:** 2025-03-05

**Authors:** Honghua Chen, Mengqian Huang, Bei Hou, Zixiang Liu, Ruyang Tan, Luna Cui, Tao Wang, Zhiyun Wang

**Affiliations:** 1 School of Life Sciences, Tianjin University 629539 , Tianjin, China; 2 School of Environmental Science and Engineering, Tianjin University 162783 , Tianjin, China; 3 Tianjin Key Laboratory of Pathogenic Microbiology of Infectious Disease, Tianjin Centers for Disease Control and Prevention 630997 , Tianjin, China; Loyola University Chicago - Health Sciences Campus, Maywood, Illinois, USA

**Keywords:** EV-D68, VP3, MAVS, NF-κB, mitochondrial damage

## Abstract

Enterovirus D68 (EV-D68) is an emerging pathogen causing severe respiratory infections, and the immune evasion mediated by EV-D68 structural protein has been under discussion for several years. Our early research has identified that EV-D68 structural protein VP3 targets specifically the interferon regulatory factor 7 to inhibit type I interferon signaling, but not interferon regulatory factor 3, which is indispensable for mitochondrial antiviral signaling protein (MAVS)-activated type I interferon signaling. Interestingly, in this study, we found that VP3 co-localizes and interacts with MAVS. Furthermore, VP3 acts as a negative regulator of MAVS/Sendai virus-activated NF-κB signaling pathway. Overexpression of VP3 can promote EV-D68 replication and reverse MAVS-mediated inhibition of virus replication. The mechanism of the interaction between VP3 and MAVS may be that VP3 not only disrupts the mitochondrial membrane potential but also leads to the release of MAVS from mitochondria. Moreover, VP3 binds to the transmembrane domain of MAVS with mitochondrial membrane localization function, which provides support for the mechanism of action. Finally, in our study, we found that VP3 interaction with MAVS to inhibit NF-κB activation is a mechanism that is prevalent in enteroviruses. Overall, our data demonstrate that the interaction between VP3 and MAVS can be used by enteroviruses to evade host innate immunity as a broad-spectrum strategy.

**IMPORTANCE:**

Enterovirus D68 (EV-D68), as an emerging pathogen, has resulted in a rising number of pediatric infections worldwide since its initial outbreak in the United States in 2014. This virus can cause severe respiratory illnesses and is linked to acute flaccid myelitis. In this article, we report that the structural protein VP3 of EV-D68 inhibits the activation of the NF-κB signaling pathway by targeting mitochondrial antiviral signaling protein (MAVS). Further studies demonstrate that VP3 can induce mitochondrial damage, resulting in the loss of MAVS localization in mitochondria. These findings suggest that the interaction between VP3 and MAVS may represent a mechanism by which EV-D68 suppresses the activation of the NF-κB signaling pathway, facilitating immune evasion and promoting viral replication. Our study suggests potential therapeutic strategies for enterovirus-related viral diseases and the development of novel antiviral drugs.

## INTRODUCTION

Enterovirus D68 (EV-D68) was initially isolated and identified from the throat swab samples of four California children suffering from pneumonia and bronchiolitis in 1962, and its main clinical symptoms are runny nose, cough, fever, and muscle aches, among others (1). In the early stage, most of the infected patients had mild symptoms, and EV-D68 used to be a rare pathogen, which did not draw enough attention from researchers (2). However, large and widespread outbreaks have been reported in Japan, China, Thailand, Italy, and the United Kingdom, among other countries, since 2005 and have resulted in deaths due to EV-D68 infection in Japan and the Philippines (3). During a large outbreak of EV-D68 in the United States in 2014, there were more than a thousand cases of children with infection (4), and some patients even suffered from acute flaccid myelitis (AFM) and central nervous system complications, such as cardiopulmonary failure, spinal cord disease, brain stem disease, limb weakness, facial nerve palsy, and dysphagia (5–7). Since then, the number of cases reported globally has also been on the increase (6, 8, 9). The outbreak of this series of epidemics worldwide and the increase in the number of EV-D68 infection cases (10), especially the number of severe patients, indicate that the epidemic intensity of the virus is increasing.

Like other enteroviruses, the EV-D68 genome consists of a single open reading frame (ORF) flanked by untranslated regions (UTRs) at both ends, which is translated into a polyprotein precursor. This ORF is translated into a polyprotein precursor, which is subsequently hydrolyzed and cleaved by its own proteases (2A^pro^ and 3C^pro^) during synthesis. This process ultimately produces 11 mature viral proteins, including four structural proteins (VP1–VP4) and seven non-structural proteins (2A^pro^, 2B, 2C, 3A, 3B, 3C^pro^, and 3D^pol^) (11, 12). The structural proteins assemble into a roughly 30 nm icosahedral capsid (13). VP1, VP2, and VP3 subunits, which have highly variable structures, form the main viral epitopes exposed to the host immune system and neutralizing antibodies (11, 14). VP4 is situated on the inner surface of the capsid (12). VP1 contains a serotype-specific neutralization site, the BC loop, making it a key target for molecular analysis, serotype differentiation, and detection of emerging strains (11), as well as an effective antiviral drug target (12). Additionally, studies have indicated that enterovirus proteins 2A^pro^ (15), 2C (16), and 3C^pro^ (17, 18) participate in the host’s interaction process and can be used as a potential antiviral drug target.

Research has shown that 3C^pro^ and 2A^pro^ of enterovirus are important molecules in the innate immune evasion strategy (19). EV-D68 3C^pro^ cleaves Toll-interleukin-1 receptor domain-containing adaptor-inducing interferon-β (17), down-regulates interferon regulatory factor 7 (IRF7) (18), inhibits the interaction of melanoma differentiation gene 5 (MDA5) with mitochondrial antiviral signaling protein (MAVS), or cleaves transforming growth factor-β-activated kinase 1 (TAK1) to inhibit the innate antiviral immune response (20). It cleaves signal transducer and activator of transcription 1 to inhibit type I interferon (IFN) signaling (21). The 2A^pro^ protein of enteroviruses exhibits a conserved function in counteracting antiviral host responses by inhibiting stress granule (SG) formation and IFN-γ gene transcription (22). Our previous studies also have shown that EV-D68 2A^pro^ inhibits antiviral type I interferon responses by cleaving tumor necrosis factor receptor-associated factor 3 (23), and EV-D68 VP3 inhibits the tumor necrosis factor receptor-associated factor 6 (TRAF6)-induced ubiquitination of IRF7 by competitive binding to IRF7 to inhibit type I interferon response (24). In addition, the structural protein VP3 of foot-and-mouth disease virus interacts with MAVS and inhibits MAVS-mediated type I interferon signaling (25). So, whether VP3 of EV68 interacts with MAVS and what effect this will have on the downstream signaling pathway of MAVS are questions we want to answer.

In this work, we found that the structural protein VP3 of EV-D68 can inhibit the phosphorylation and nuclear entry of p65 in the NF-κB signaling pathway in infected cells, and this is closely related to the interaction between VP3 and MAVS. In addition, we found that VP3 targets the transmembrane (TM) domain of MAVS and leads to the release of MAVS from mitochondria. Moreover, it is noteworthy that VP3 of other enteroviruses, such as enterovirus A71 (EV-A71) and coxsackievirus A16 (CV-A16), can also interact with MAVS and inhibit the NF-κB signaling pathway. In summary, these results provide new evidence that VP3 regulates the NF-κB signaling pathway, and this may be a broad-spectrum strategy used by enteroviruses to evade host innate immunity.

## RESULTS

### VP3 inhibits MAVS-induced NF-κB signaling

**Fig 1:**
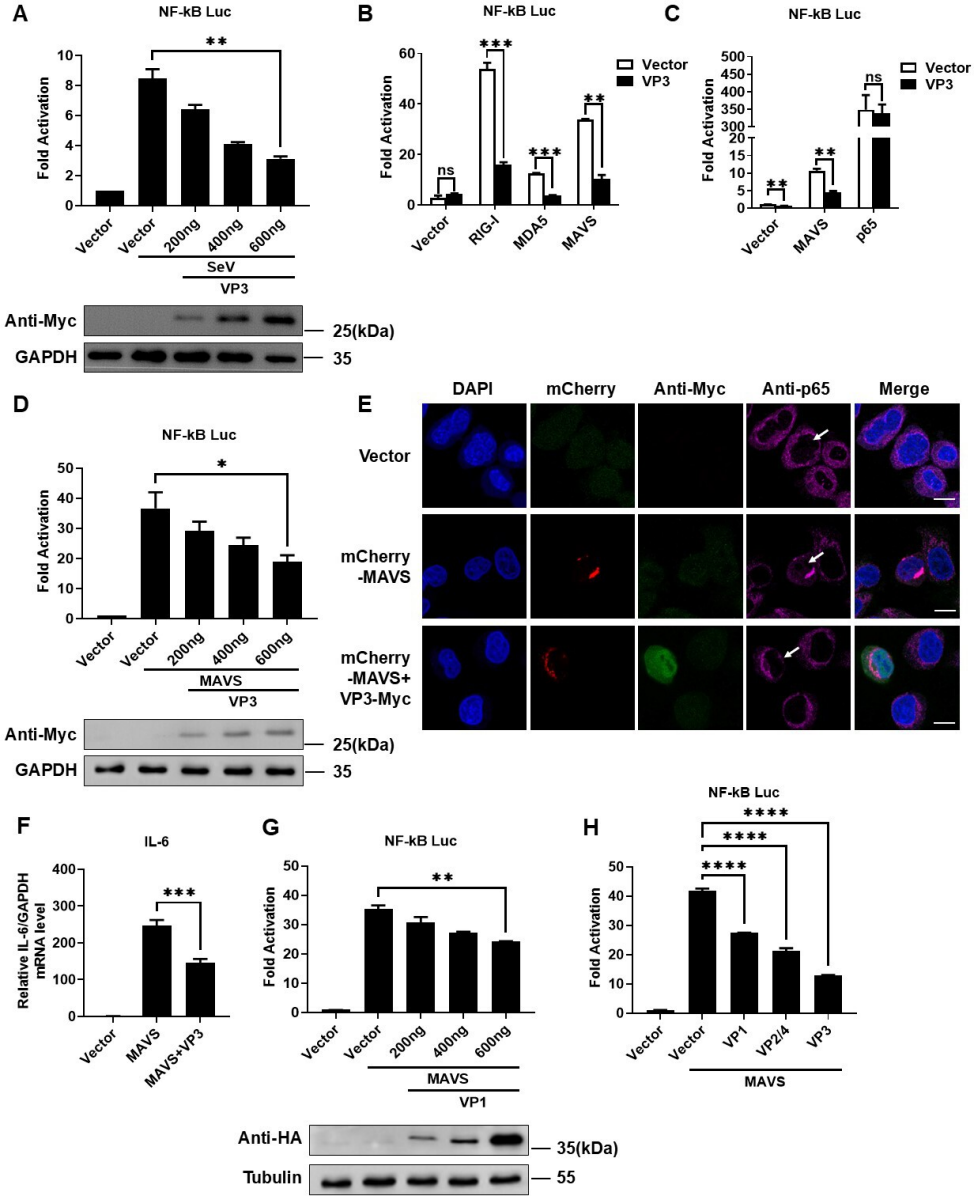
EV-D68 VP3 inhibits MAVS-induced NF-kB signal. (**A**) 300 ng NF-κB Luc reporter, 1 ng pRL-SV40 plasmid co-transfected with gradient doses VP3-Myc (0, 200, 400, and 600 ng) into HEK293T cells. After 24 h of transfection, SeV infected the cells for 16 h. Cell lysates were harvested for dual-luciferase reporter assay and western blot. (**B**) 300 ng NF-κB Luc, 1 ng pRL-SV40, and 600 ng VP3-HA were co-transfected with 200 ng Vector, RIG-I, MDA5, or MAVS plasmids into HEK293T, and dual-luciferase reporter assay was performed after 24 h of transfection. (**C**) 300 ng NF-κB Luc, 1 ng pRL-SV40, and 600 ng VP3-HA were co-transfected with 200 ng Vector, MAVS, or p65 plasmids into HEK293T, and dual-luciferase reporter assay was performed after 24 h of transfection. (**D**) HEK293T cells were co-transfected with 300 ng NF-κB luc, 1 ng pRL-SV40, 200 ng MAVS plasmids, and gradient doses of VP3-Myc plasmids (0, 200, 400, and 600 ng). After 24 h, cells were harvested for dual-luciferase reporter assay and western blot analysis. (**E**) HeLa cells were co-transfected with 500 ng mCherry-MAVS and VP3-Myc plasmids (Vector plasmids as control). After 24 h, the cells were fixed for immunofluorescence staining. mCherry-MAVS (red), VP3-Myc (green), p65 (purple), and nuclei (blue, stained with DAPI) were visualized. Scale bar: 20 µm. (**F**) RD cells were co-transfected with Vector, MAVS, or MAVS + VP3. After 36 h, cells were collected for extracting total RNA, and the expression of IL-6 mRNA was determined by RT-qPCR assay. Results are expressed as mRNA levels relative to GAPDH mRNA levels. (**G**) HEK293T cells were co-transfected with 300 ng NF-κB luc, 1 ng pRL-SV40, 200 ng MAVS plasmid, and gradient doses of VP1-HA plasmid (0, 200, 400, and 600 ng). 24 h after transfection, cells were collected for dual-luciferase reporter assay and western blot analysis. (**H**) HEK293T cells were co-transfected with 300 ng NF-κB luc, 1 ng pRL-SV40, 200 ng MAVS plasmid, and 600 ng Vector, VP1-HA, VP2/4-HA, or VP3-HA plasmid. After 24 h, cells were collected for dual-luciferase reporter assay.

Since VP3 can influence the type I interferon pathway activated by IRF7 but not IRF3 (24), we aimed to investigate whether other antiviral innate immune responses downstream of retinoic acid-inducible gene I (RIG-I)-like receptor (RLR) signaling, particularly the NF-κB pathway, might also be affected. Using SeV as an activator of the NF-κB pathway, we found that VP3 inhibited the activation of the NF-κB signaling pathway (Fig. 1A). Consistent with this, the dual-luciferase reporter assay showed that EV-D68 VP3 blocked the activation of the NF-κB promoter mediated by RIG-I, MDA5, MAVS, and p65 (Fig. 1B and C), which indicated that EV-D68 VP3 may inhibit the activation of NF-κB by interfering with MAVS signaling. In order to further confirm this conclusion, we activated NF-κB signaling using MAVS and subsequently found that VP3 had a gradient inhibitory effect on the activity of the NF-κB promoter activated by MAVS (Fig. 1D). Additionally, we found that EV-D68 VP3 can inhibit the nuclear translocation of p65 and the expression of downstream antiviral genes (Fig. 1E and F; Fig. S1A and S1B).

In this study, we utilized the EV-D68 VP1 protein as a control to compare the effects of VP1 and VP3 on MAVS. The results of the dual-luciferase reporter assay showed that VP1 could inhibit the MAVS-activated NF-κB promoter activity (Fig. 1G). However, when comparing the inhibitory effects of other structural proteins and VP3 on MAVS-activated NF-κB promoter activity, it was found that VP3 had a 70% inhibitory effect, while VP1 had a 35% inhibitory effect, and VP2/4 had a 50% inhibitory effect (Fig. 1H). In conclusion, compared to VP1 and VP2/4, VP3 plays a pivotal role in suppressing the MAVS-mediated NF-κB signaling pathway. In order to further explore the biological significance of the interaction between EV-D68 VP3 and MAVS, we assessed the effect of VP3 on virus replication by RT-qPCR. The results showed that in the process of EV-D68 [multiplicity of infection (MOI) = 0.1] infection (6, 12, and 24 h), the transfection of VP3 promoted virus replication significantly (Fig. S2A). Next, we detected the effect of MAVS or MAVS + VP3 on virus replication, and the results showed that overexpression of MAVS inhibited the replication of virus, and the presence of VP3 reversed the effect of MAVS on inhibition of virus (Fig. S2B).

### EV-D68 VP3 induces MAVS release from the mitochondria

**Fig 2:**
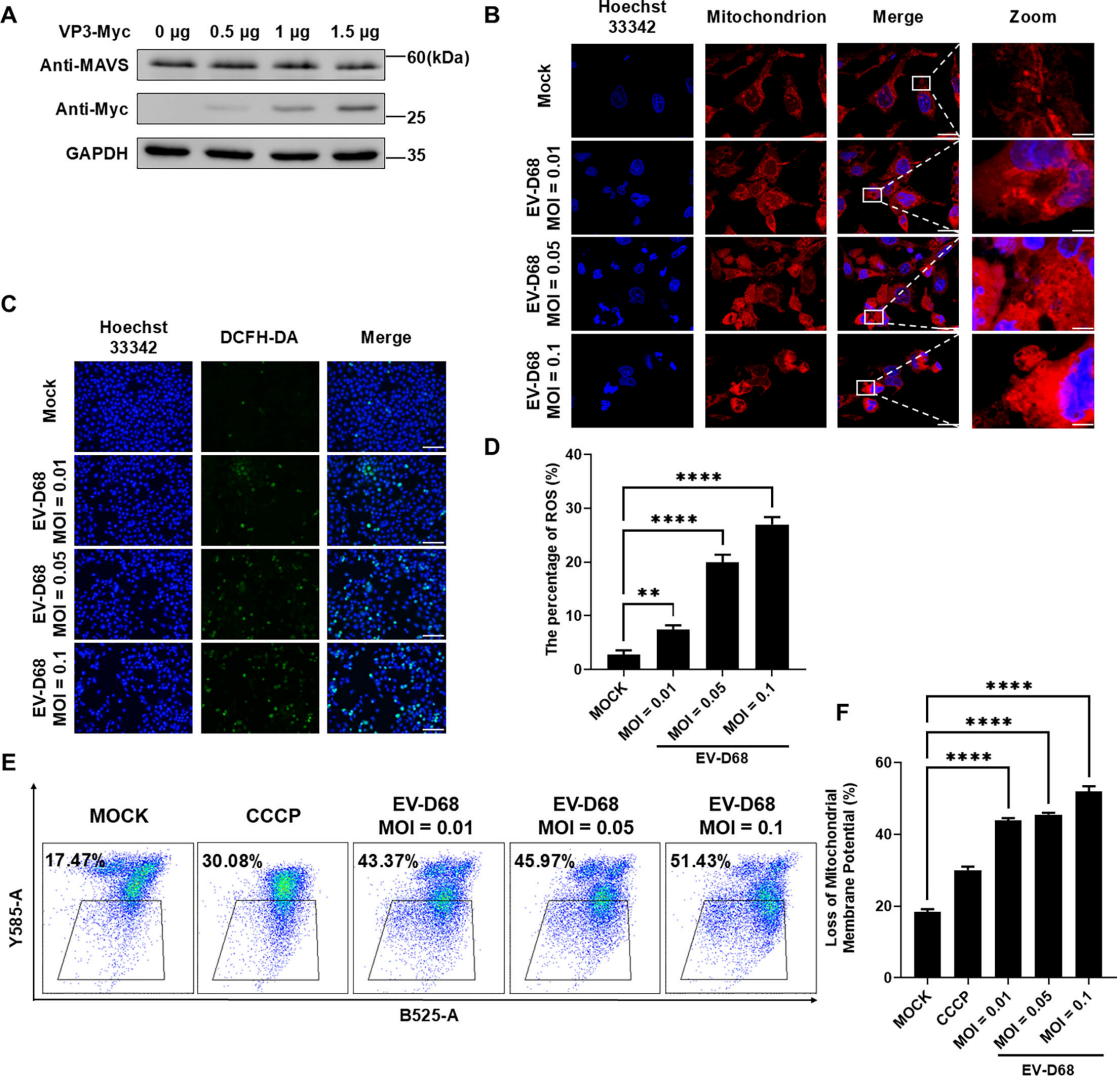
EV-D68 infection causes mitochondrial abnormalities. (**A**) HEK293T cells were transfected with VP3-Myc plasmids (0, 0.5, 1, and 1.5 µg) and collected 24 h later. Cell lysates were analyzed by western blotting with anti-MAVS and anti-Myc tagged antibodies. GAPDH served as an internal control. (**B**) RD cells were infected with EV-D68 at different MOIs (MOI = 0.01, 0.05, 0.1). MitoTracker Red CMXRos mitochondrial red fluorescent probe (500 nM) and Hoechst dye were added after 24 h and incubated at 37°C for 30 min. Cells were observed using a confocal microscope. Scale bar: 20 (Merge) and 3.5 µm (Zoom). (**C**) HeLa cells were infected with EV-D68 at different MOIs (MOI = 0.01, 0.05, 0.1). DCFH-DA probe (1:3,000) and Hoechst dye (1:100) were added after 24 h and incubated at 37°C for 30 min. Cells were observed using fluorescence microscopy. Scale bar: 100 µm. (**D**) The percentage of ROS was quantified by ImageJ. (**E**) RD cells were infected with EV-D68 at different MOIs (MOI = 0.01, 0.05, 0.1). Cells were incubated with JC-10 at 37°C for 20 min. The proportion of cells with reduced mitochondrial membrane potential was detected using flow cytometry. (**F**) Quantified data of loss of mitochondrial membrane potential in **E**.

Studies have shown that various viruses evade the host’s antiviral immune response by degrading MAVS (25–27). Given that enterovirus 2A^pro^ can cleave MAVS (28), we hypothesized whether VP3 has a similar ability to degrade MAVS. However, our results indicated that the EV-D68 VP3 protein did not cause a significant degradation of MAVS (Fig. 2A; Fig. S3A). In addition, factors that affect the physical state of mitochondria, such as mitochondrial fusion, changes in membrane potential, and reactive oxygen species (ROS) levels, can also change the formation of MAVS aggregates (29–31). During EV-D68 infection, significant cytopathic effects and cell apoptosis occur, which may lead to mitochondrial damage that interferes with MAVS function. Related studies on mitochondria had shown that the EV-D68 infection process will cause the destruction of mitochondrial morphology (Fig. 2B), which may be linked to EV-D68’s ability to cause apoptosis. ROS and mitochondrial membrane potential serve as important retrograde signal reflecting the functional status of mitochondria (32). The data showed that the level of cellular ROS increased (Fig. 2C and D) and the mitochondrial membrane potential decreased (Fig. 2E and F) with the increase in the EV-D68 infection dose.

**Fig 3:**
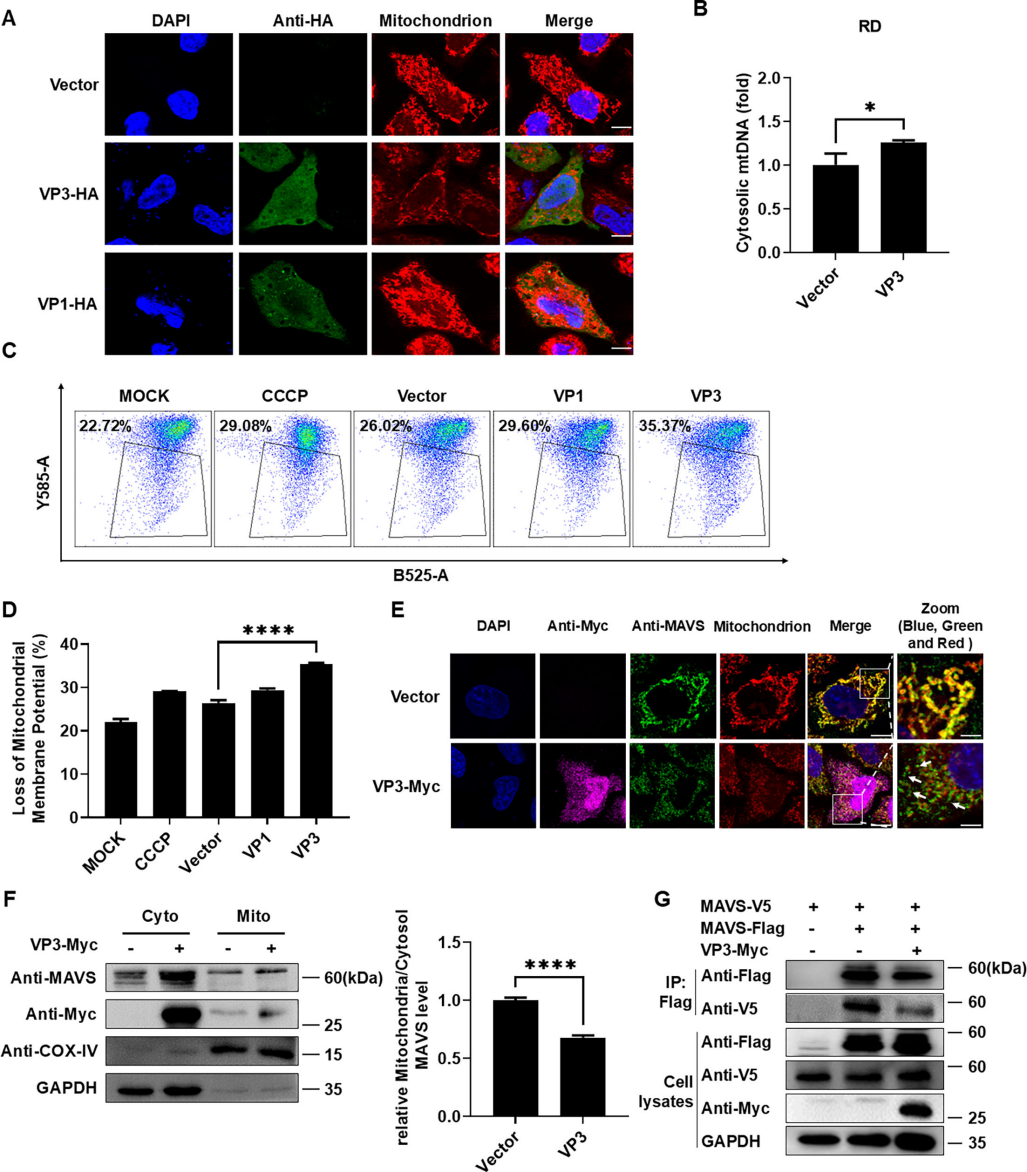
EV-D68 VP3 induces MAVS leakage from the mitochondria. (**A**) HeLa cells were transfected with 500 ng vector, VP1-HA, or VP3-HA plasmid, and MitoTracker Red CMXRos mitochondrial red fluorescent probe (500 nM) was added after 24 h, and then incubated at 37°C for 30 min. Cells were fixed and stained with immunofluorescence. Mitochondria (red), VP1-HA or VP3-HA (green), and nuclei (blue, stained with DAPI) were visualized. Scale bar: 20 µm. (**B**) RD cells were transfected with vector or VP3, and the mitochondrial DNA (mtDNA) copy number was detected by RT-qPCR 24 h after the transfection. The results were shown as the change of the mitochondrial DNA copy number in the transfected VP3 group relative to the vector group and normalized by RNA 18S rDNA. (**C**) RD cells were transfected with vector, VP3, or VP1. The proportion of cells with reduced mitochondrial membrane potential was detected using flow cytometry. (**D**) The quantified data of loss of mitochondrial membrane potential in C. (**E**) HeLa cells were transfected with 500 ng vector or VP3-Myc and fixed 24 h later. Corresponding immunofluorescence staining was performed. Mitochondria (red), MAVS (green), VP3-Myc (purple), and nuclei (stained with DAPI, blue) were visualized. The mitochondria (red), MAVS (green), and nuclei (stained with DAPI, blue) are shown in Zoom without the presence of VP3-Myc (purple). Scale bar: 10 (Merge) and 2.5 µm (Zoom). (**F**) RD cells were transfected with 6 µg VP3-Myc or Vector. 36 h after, following cell collection, mitochondrial and cytoplasmic fractions were isolated and subjected to western blot analysis. GAPDH and COX-IV were used as internal references for the cytoplasm and mitochondria, respectively. The gray levels of MAVS, GAPDH, and COX-IV were quantified using ImageJ. (**G**) HEK293T cells were transfected with 2 µg MAVS-Flag, 2 µg MAVS-V5, and 2 µg VP3-Myc plasmids (vector was transfected as controls). After 48 h of transfection, the cell lysates were incubated with Anti-Flag M2 Affinity Gel for co-immunoprecipitation experiments, and the levels of MAVS-Flag, MAVS-V5, and VP3-Myc proteins in the samples were analyzed by western blot. GAPDH served as an internal reference.

Interestingly, when the Vector or VP3 plasmid was transfected into HeLa cells and subjected to mitochondrial and immunofluorescence staining, the results showed that in the cells of the Vector group, mitochondria exhibited the characteristic short, rod-shaped morphology, but in the experimental group transfected with VP3, mitochondrial morphology was significantly altered, with notable changes in red fluorescent foci, while green fluorescent foci confirmed the expression of the VP3 protein in the cells. This result suggested that overexpression of VP3 affects mitochondria. To further strengthen the validity of this conclusion, EV-D68 VP1 protein was selected as a control to conduct the same experiment. After transfection of the EV-D68 VP1 plasmid, the mitochondrial fluorescence foci in the cells did not change significantly, indicating that EV-D68 VP1 protein did not affect mitochondria (Fig. 3A). However, RT-qPCR results showed that VP3 slightly facilitated the release of mitochondrial DNA (mtDNA) (Fig. 3B). Subsequently, flow cytometry was used to assess the effect of VP3 on mitochondrial membrane potential, with carbonyl cyanide m-chlorophenyl hydrazone serving as a positive control for inducing mitochondrial depolarization. The results demonstrated that the proportion of cells with reduced mitochondrial membrane potential in the VP3 overexpression group was approximately one-third higher compared to that of the transfected Vector or VP1 group (Fig. 3C and D). Taken together, EV-D68 VP3 disrupts mitochondrial membrane potential and thus affects mitochondria.

Since MAVS is primarily localized to the mitochondrial outer membrane, and its proper localization is required for its function (33, 34), we sought to determine whether VP3 affects the distribution of MAVS within the mitochondria. To address this, we performed an immunofluorescence assay. The results are shown in Fig. 3E. In the Vector transfection group, the cells exhibited a uniform linear filamentous morphology, and it was observed that MAVS was localized on mitochondria. However, in the VP3-transfected group, mitochondrial fragmentation and punctate structure were observed, and MAVS was released from mitochondria and lost its co-localization with mitochondria. We also transfected the RD cells with VP3 or Vector and isolated the mitochondrial component for western blot analysis. The results demonstrated increased release of MAVS from the mitochondria into the cytosol in the VP3-transfected group (Fig. 3F). In summary, EV-D68 VP3 affects the localization of MAVS in the mitochondria, causing it to be released from the mitochondria. Furthermore, we evaluated the effect of EV-D68 VP3 on MAVS aggregation by co-transfecting HEK-293T cells with MAVS-Flag, MAVS-V5, and VP3-Myc plasmids for co-immunoprecipitation experiments. The results demonstrated that VP3 expression led to a decrease in MAVS aggregation (Fig. 3G; Fig. S3B). These findings further indicate that the EV-D68 VP3 protein can inhibit the activation of MAVS and downstream NF-κB signaling.

### Interaction patterns between EV-D68 VP3 and MAVS

**Fig 4:**
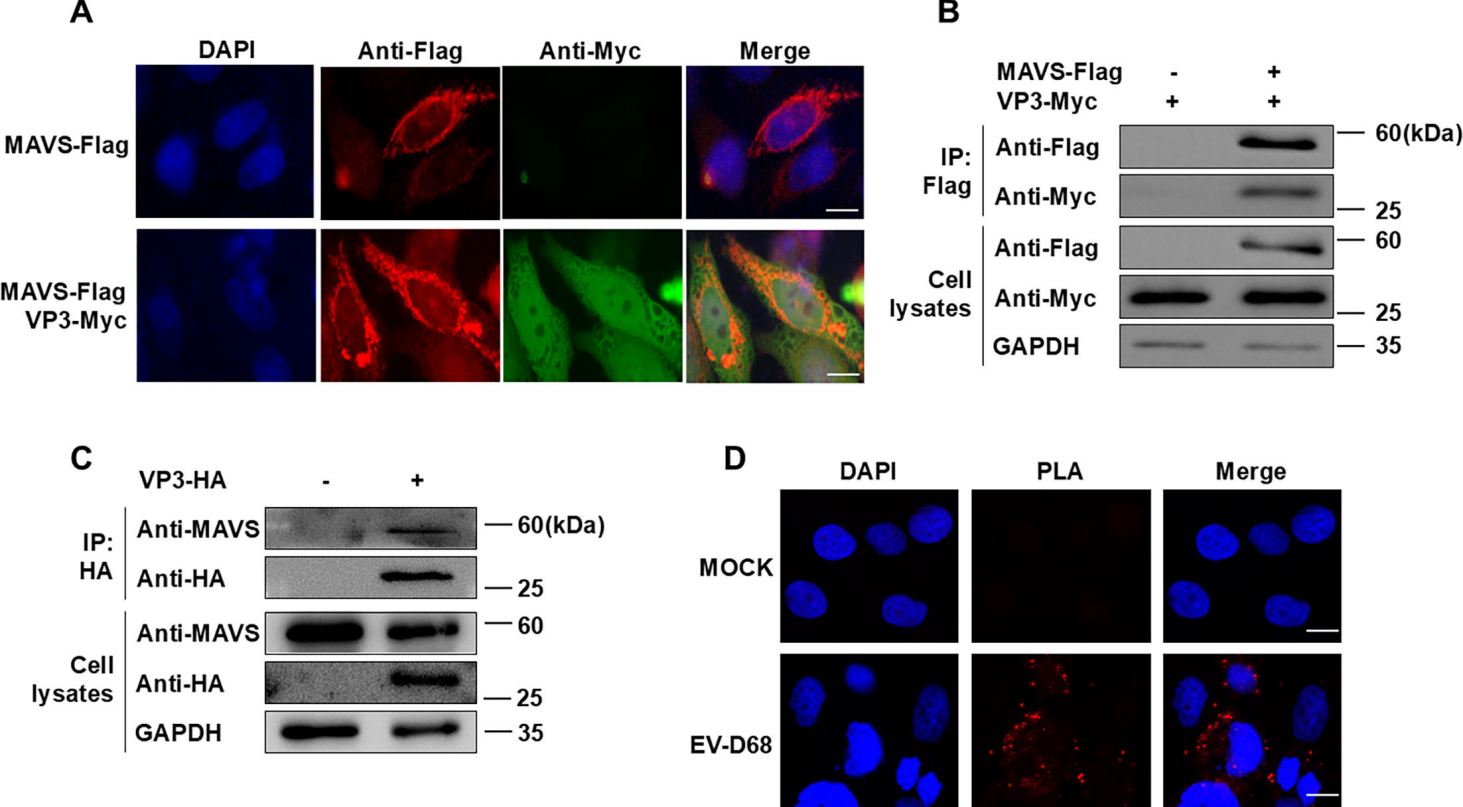
EV-D68 VP3 interacts with MAVS. (**A**) HeLa cells were co-transfected with 500 ng MAVS-Flag and VP3-Myc plasmids (Vector plasmids as control). After 24 h, the cells were fixed for immunofluorescence staining. MAVS-Flag (red), VP3-Myc (green), and nuclei (stained with DAPI, blue) were visualized. Scale bar: 20 µm. (**B**) HEK293T cells were transfected with 4 µg VP3-Myc and 4 µg MAVS-Flag plasmids (Vector plasmids as control). After 48 h of transfection, the cell lysates were incubated with Anti-Flag M2 Affinity Gel for co-immunoprecipitation experiments, and the levels of MAVS and VP3 proteins in the samples were analyzed by western blot. GAPDH served as an internal reference. (**C**) HEK293T cells were transfected with 8 µg VP3-HA plasmids (Vector plasmids as control). After 48 h of transfection, the cell lysates were incubated with Anti-HA Affinity Matrix for co-immunoprecipitation experiments, and the levels of MAVS and VP3 proteins in the samples were analyzed by western blot. GAPDH served as an internal reference. (**D**) HeLa cells were infected with EV-D68 (MOI = 0.1) or not for 24 h. The interaction of VP3 and MAVS in HeLa cells was determined by PLA. Scale bar: 20 µm.

Furthermore, we aimed to clarify how EV-D68 affects MAVS functionality. First, we observed the localization of MAVS and VP3 proteins. Transient transfection of MAVS and VP3 plasmids revealed that MAVS and VP3 had a co-localization phenomenon (Fig. 4A). Co-immunoprecipitation experiment and the proximity ligation assay (PLA) demonstrated an interaction between VP3 and MAVS (Fig. 4B through D).

**Fig 5:**
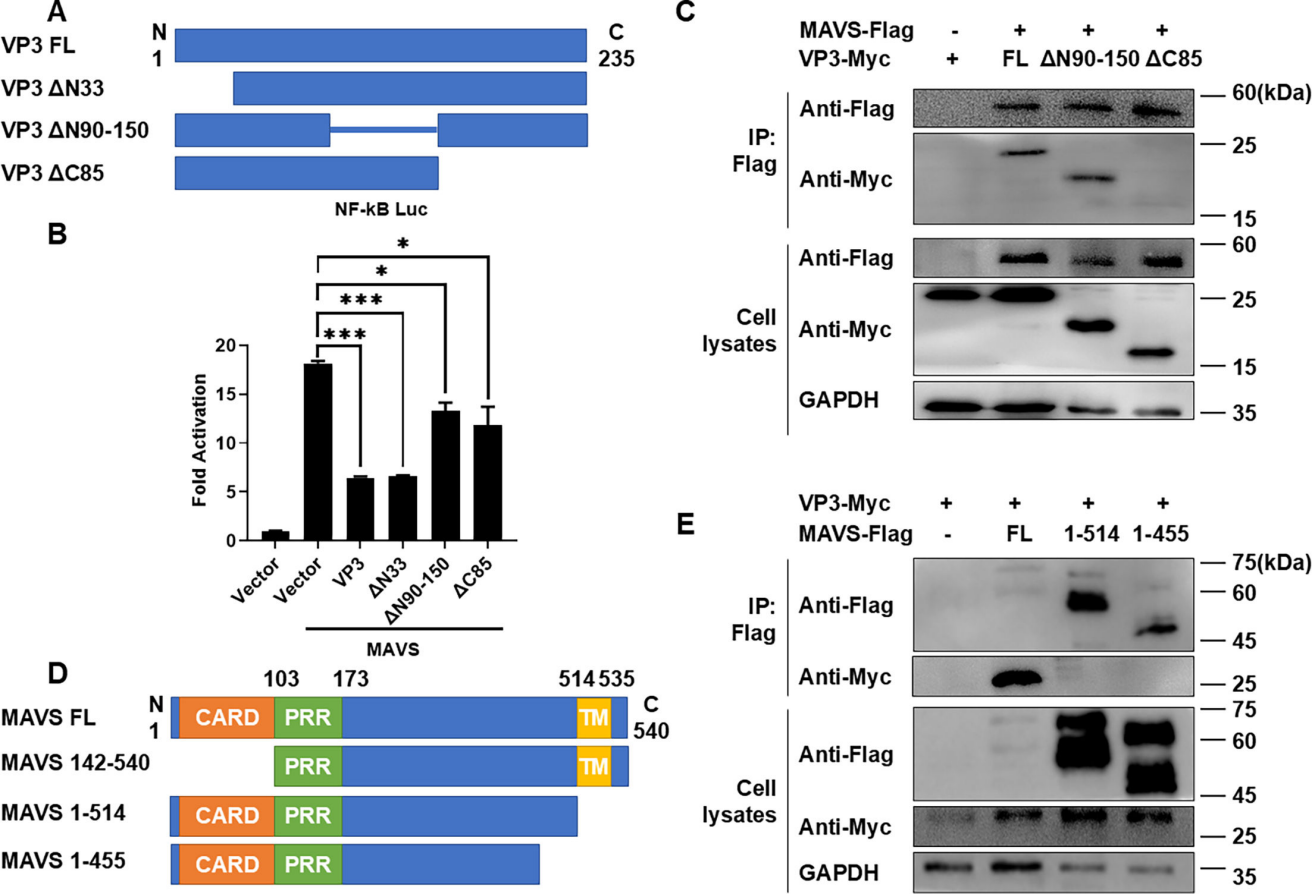
Interaction site of EV-D68 VP3 with MAVS. (**A**) Diagrams of full-length and truncated VP3. (**B**) 300 ng NF-κB Luc, 1 ng pRL-SV40, and 300 ng MAVS plasmid were co-transfected with 500 ng VP3-Myc or its truncated plasmids into HEK293T cells, and the cell lysates were collected after 24 h for dual-luciferase reporter assay. (**C**) 3 µg MAVS plasmid was co-transfected with 3 µg Vector, VP3 FL-Myc, VP3 ΔN90-150-Myc, or VP3 ΔC85-Myc into HEK293T cells. After 48 h of transfection, the cell lysates were incubated with Anti-Flag M2 Affinity Gel for co-immunoprecipitation experiments, and the levels of MAVS and VP3 truncation levels in samples were analyzed by western blot. GAPDH served as an internal reference. (**D**) Diagrams of full-length and truncated MAVS. (**E**) 3 µg VP3-Myc plasmid was co-transfected with 3 µg Vector, MAVS FL-Flag, MAVS 1–514-Flag, or MAVS 1–455-Flag into HEK293T cells. Cells were harvested after 48 h for co-immunoprecipitation experiments, and the levels of MAVS truncation and VP3 levels in samples were analyzed by western blot. GAPDH served as an internal reference.

In order to further determine the interaction mechanism between EV-D68 VP3 and MAVS, a series of VP3 truncations were constructed and co-transfected with MAVS plasmids into HEK293T cells. The dual-luciferase reporting experiment results showed that, compared with the full length of EV-D68 VP3, the two truncated mutants of EV-D68 VP3ΔN90-150 and EV-D68 VP3ΔC85 exhibited a substantial diminution in their capacity to inhibit the activity of the NF-κB promoter activated by MAVS (Fig. 5A and B). However, co-immunoprecipitation experiments found that the interaction between the VP3ΔC85 and MAVS plasmids was significantly weakened, whereas no such reduction was observed between the VP3ΔN90-150 and MAVS plasmid (Fig. 5C; Fig. S4A).

MAVS is composed of 540 amino acids. The N-terminal caspase-recruitment domains (CARD) interact with the CARD domain of RIG-I/MDA5, and the C-terminal is the TM domain, positioning MAVS on the outer mitochondrial membrane. Additionally, the C-terminal amino acids 455–460 are necessary for TRAF6-mediated activation of the NF-κB pathway (35, 36). According to the functional domain of MAVS, three truncated mutants were constructed (Fig. 5D) and co-transfected with EV-D68 VP3 plasmid for immunoprecipitation experiments. It was found that MAVS 1–514 and MAVS 1–455 lost the interaction with VP3 (Fig. 5E; Fig. S4B through D), which means that the TM domain of MAVS is the essential site for the interaction with EV-D68 VP3. This can also well explain the reason that VP3 inhibits the NF-κB signaling pathway activated by MAVS and causes the release of MAVS from the mitochondria.

### The interaction between VP3 and MAVS is universal in the enteroviruses

**Fig 6:**
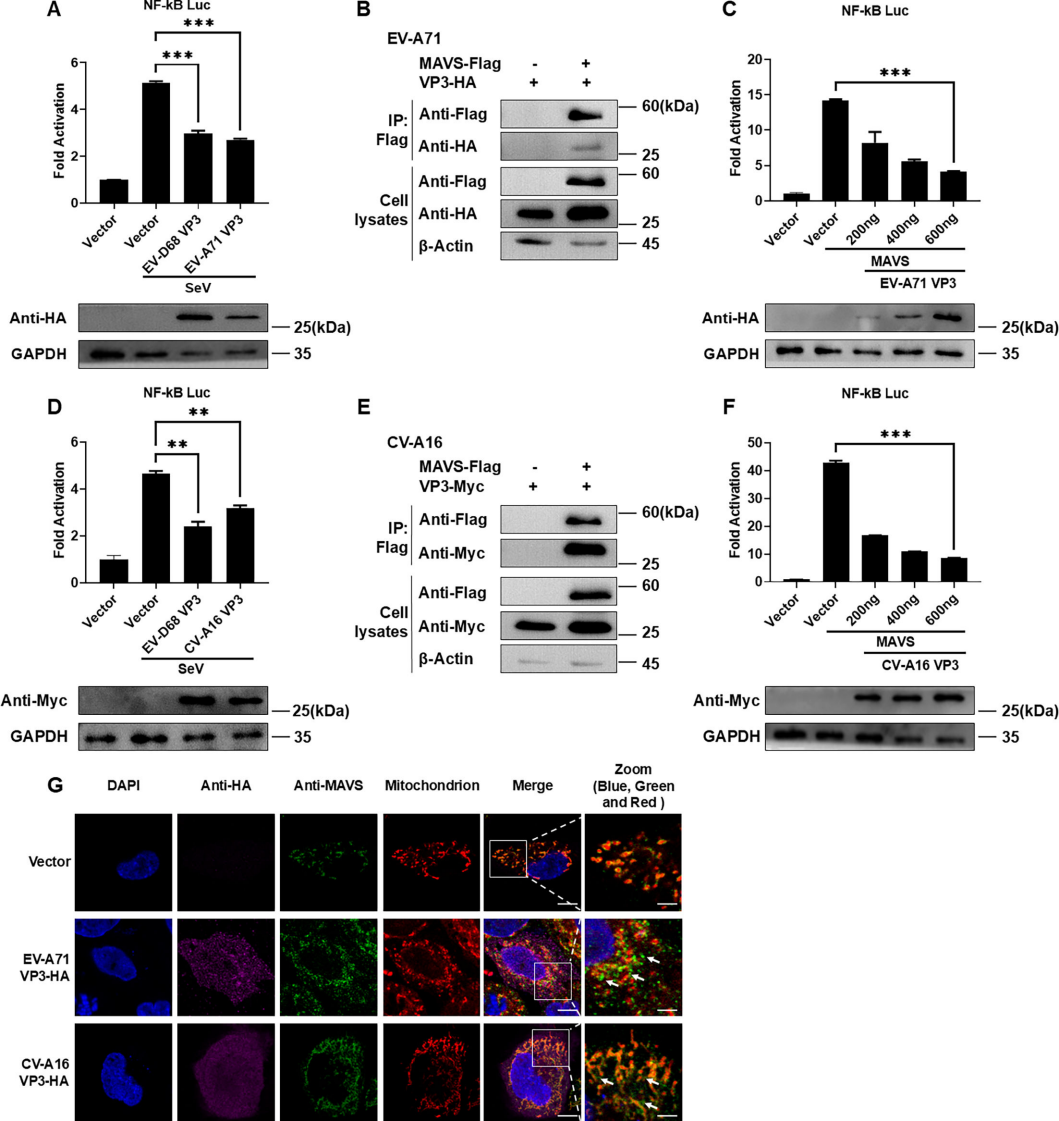
EV-A71 and CV-A16 VP3 interact with MAVS and inhibit the NF-κB signaling. (**A, D**) 300 ng NF-κB Luc and 1 ng pRL-SV40 were co-transfected with 500 ng Vector, EV-D68 VP3-HA, or EV-A71 VP3-HA (**A**), EV-D68 VP3-Myc, or CV-A16 VP3-Myc (**D**) into HEK293T cells. After 24 h, cells were infected with SeV for 16 h. The cells were harvested and subjected to dual-luciferase reporter assay and western blot analysis. (**B, E**) 3 µg EV-A71 VP3-HA (**B**) or 3 µg CV-A16 VP3-Myc (**E**) plasmids were co-transfected with 3 µg MAVS-Flag into HEK293T cells. After 48 h, the cell lysates were incubated with Anti-Flag M2 Affinity Gel for co-immunoprecipitation experiments. The levels of MAVS and VP3 in the samples were analyzed by western blot. β-Actin was used as an internal reference. (**C, F**) 300 ng NF-κB Luc, 1 ng pRL-SV40, and 300 ng MAVS plasmid were co-transfected with gradient doses of EV-A71 VP3-HA plasmid (0, 200, 400, and 600 ng) (**C**) or CV-A16 VP3-Myc plasmid (0, 200, 400, and 600 ng) (**F**) into HEK293T cells, and cell lysates were harvested after 24 h for dual-luciferase reporter assay and western blot analysis. (**G**) 500 ng Vector, EV-A71 VP3-HA plasmid, or CV-A16-HA plasmid was transfected into HeLa cells. After 24 h, the cells were fixed for immunofluorescence staining. Mitochondria (red), MAVS (green), VP3-HA (purple), and nuclei (stained with DAPI, blue) were visualized. The mitochondria (red), MAVS (green), and nuclei (stained with DAPI, blue) are shown in Zoom without the presence of VP3-HA (purple). Scale bar: 10 (Merge) and 2.5 µm (Zoom).

In order to further explore whether the interaction between VP3 and MAVS is unique to EV-D68 or widespread in the enteroviruses, EV-A71 VP3 and CV-A16 VP3 plasmids were constructed. Through dual-luciferase reporter and immunoprecipitation experiments, it was found that, same as EV-D68 VP3, EV-A71 VP3 and CV-A16 VP3 can inhibit the activity of NF-κB promoter activated by SeV (Fig. 6A and D) and interact with MAVS (Fig. 6B and E). The EV-A71 VP3 and CV-A16 VP3 plasmids were transfected with gradient amounts, and these two plasmids had a gradient inhibitory effect on the activity of the NF-κB promoter activated by MAVS (Fig. 6C and F). Immunofluorescence assays showed that VP3 from both EV-A71 and CV-A16 induces MAVS dissociation from mitochondria, thereby disrupting its normal function (Fig. 6G). The above results indicate that the interaction between VP3 and MAVS is widespread in the enteroviruses, which may be an important way to antagonize host innate immune responses following enterovirus infection.

## DISCUSSION

**Fig 7:**
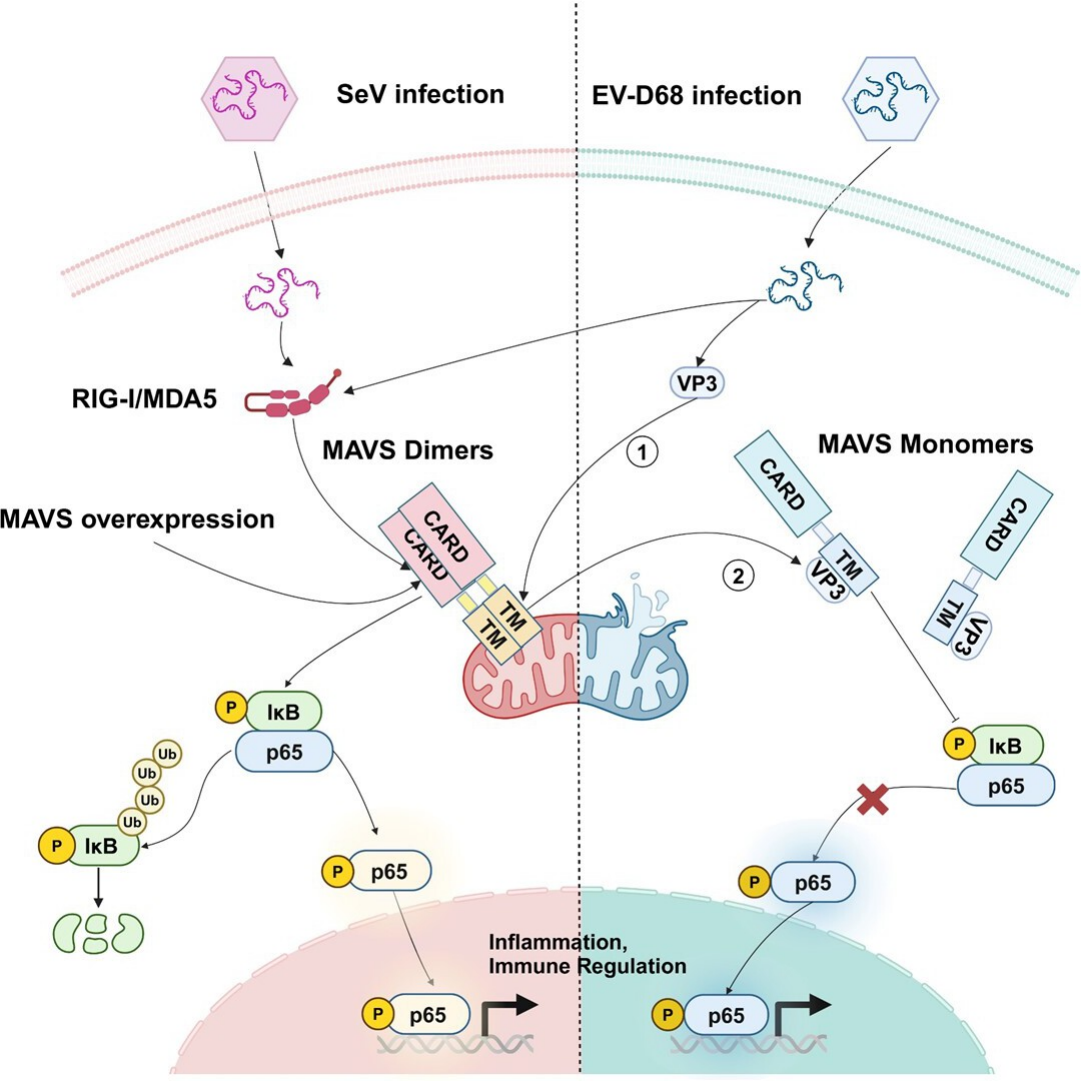
Proposed model of the interaction between EV-D68 VP3 and MAVS. When cells are infected with SeV, or when MAVS is overexpressed in cells, MAVS is activated to initiate a series of downstream signals and interfere with viral replication. After the EV-D68 virus enters cells, it is recognized by RIG-I-like receptors and transmits signals to MAVS. The interaction of the structural protein VP3 of EV-D68 with MAVS includes: (1) VP3 of EV-D68 can bind to the TM domain of MAVS and induce MAVS leakage from the mitochondria; and (2) VP3 negatively regulates the NF-κB signaling pathway downstream of MAVS. RIG-I, retinoic acid-inducible gene I; MDA5, melanoma differentiation-gene 5; MAVS, mitochondrial antiviral signaling protein; NF-κB, nuclear factor kappa-B; IκB, inhibitor of NF-κB. The image was created with BioRender.com.

EV-D68 is associated with mild to severe acute respiratory illness (ARI), acute flaccid myelitis (AFM), and acute flaccid paralysis (AFP) (37, 38). However, there are currently no effective vaccines or antiviral drugs for EV-D68 (12), and the infection and pathogenic mechanisms of EV-D68 are incompletely understood. Previous studies have shown that non-structural proteins of EV-D68, especially 3C^pro^ and 2A^pro^, are important molecules for immune escape, but as viral structural proteins that appear earlier in the process of viral infection, their role in escaping the innate immune response of the virus is not fully clear. Recent research by our group has shown that VP3 suppresses TRAF6-induced ubiquitination of IRF7 to inhibit IRF7-mediated type I interferon production without affecting IRF3 (24). However, in this study, we found that VP3 interacts with MAVS and for another pathway downstream of MAVS, the NF-κB signaling pathway, has a significant negative regulatory effect and promotes virus replication (Fig. 7). This may be an important mechanism of EV-D68 to evade the host innate immunity and establish rapid infection.

The innate immune system is an essential line of defense against enterovirus infection, which enables innate immune cells to respond quickly to invading viruses and induce multiple responses to interfere with the growth of the virus after identifying pathogens through specific pathogen-associated molecular patterns (39, 40). The NF-κB signaling pathway is a central regulator in innate immunity. In order to establish infection, during the co-evolution of the virus and the host, the virus has developed a variety of strategies to evade the host innate immune response, which can be divided into targeted signaling molecules to actively inhibit the formation of signal complexes or the expression of cellular proteins, the isolation or relocation of RLRs, the manipulation of the formation of SG, the cleavage of innate immune cytokines, and the manipulation of the post-translational modification of innate immune signaling pathways, receptors, and downstream signaling molecules, etc. (41–43). MAVS is the signal transduction center initiated by RIG-I-like receptors. Increasing evidence suggests that viruses can evade the host’s antiviral response by interfering with multiple points in the MAVS signaling pathway (27). The RIG-I/MAVS pathway is significantly affected during human papillomavirus and dengue virus (DENV) infection (44, 45). MAVS is cleaved by enterovirus non-structural proteins 3C^pro^ and 2A^pro^ (20, 28, 46). However, our study finds the interaction between EV-D68 structural protein VP3 and MAVS, and this interaction has also been confirmed in EV-A71 and CV-A16, providing significant insights into the immune evasion mechanisms of enteroviruses. Moreover, this study found that the interaction of EV-D68 VP3 with MAVS resulted in the release of MAVS from mitochondria, which may be an important supplement to the mechanism of action of enteroviruses in regulating MAVS. Concordantly, the overexpression of MAVS could inhibit the replication of EV-D68, which has been confirmed in CV-A16 (47). Interestingly, the overexpression of VP3 can promote virus replication and reverse the inhibitory effect of MAVS on the virus, suggesting that VP3 plays a critical role in sustaining viral infection and may function similarly in related viruses.

Since the discovery of MAVS, the role of mitochondria in antiviral innate immunity has gradually gained attention (48). Mitochondria provide a platform for hepatitis A virus, hepatitis C virus , and EV-A71 viral protein to cleave MAVS (49–51). DENV regulates mitochondrial morphology by cleaving mitochondrial fusion proteins by NS2B3 protease for infection (52). The influenza virus protein PB1-F2 inhibits the induction of type I interferon by binding to the MAVS TM domain and reducing the mitochondrial membrane potential (53). Although there have been reports about the EV-A71 infection inducing mitochondrial ROS generation to promote viral replication (54), the underlying mechanisms remain unclear. It has also been reported that EV-A71 non-structural protein 2BC localizes on mitochondria and is capable of inducing mitochondria clustering (55, 56). In this study, we first found that the cellular ROS level increases with the increase in the EV-D68 infection dose and causes mitochondrial abnormalities (Fig. 2). Additionally, transient transfection of the EV-D68 structural protein VP3 reduces the mitochondrial membrane potential (Fig. 3), which may be similar to the role of influenza virus protein PB1-F2 (53). In conclusion, although the interaction mechanism between EV-D68 VP3 and mitochondria still needs more experimental evidence to explore, the existing phenomenon also deserves researchers' further attention to the changes in mitochondria during the life cycle of *Picornaviridae* to further understand virus–host interaction.

Overall, our results indicate that EV-D68 VP3 interacts with MAVS, which activates NF-κB and IFN signaling (57), as a strategy to escape the host innate immunity. Our research group recently discovered that VP3 targets IRF7 to inhibit IRF7-mediated type I interferon production, but not IRF3 (24). Therefore, the interaction of VP3 and MAVS may be more inclined to inhibit the NF-κB signaling pathway to promote viral replication. But, whether EV-D68 VP3 affects MAVS function independently of blockade of IRF7 function continues to be explored. In this study, we also identified that VP3 binds to the TM domain of MAVS, thereby affecting MAVS’s localization and mitochondrial membrane potential. However, it is not clear whether the two effects caused by VP3 are related to each other or separate from each other. Finally, we found that the interaction between VP3 and MAVS also exists in EV-A71 and CV-A16. Therefore, our findings reveal a new mechanism of enteroviruses for evading the host innate immunity mediated by viral structural protein, which is of great significance for determining new treatment and prevention regimens.

## MATERIALS AND METHODS

### Viruses and cell lines

The EV-D68 Fermon strain (GenBank accession no. KU844179.1) was obtained from Xiaofang Yu (Cancer Center, Zhejiang University) and propagated in Rhabdomyosarcoma (RD) cells. The SeV BB1 strain was kindly provided by Lishu Zheng (Chinese Centers for Disease Control and Prevention, Beijing, China). RD cells [CRL-136; American-Type Culture Collection (ATCC), Manassas, VA, USA], human embryonic kidney 293T (HEK293T) cells (CRL-11268; ATCC), and Henrietta Lacks (HeLa) cells (CCL-2; ATCC) were cultured in Dulbecco’s modified Eagle’s medium supplemented with 10% fetal bovine serum and 1% penicillin–streptomycin (100 IU/mL) at 37°C in a 5% CO_2_ humidified atmosphere.

### Antibodies and reagents

NF-κB p65 monoclonal antibody (66535-1-Ig), MAVS; VISA polyclonal antibody (14341-1-AP), MAVS; VISA monoclonal antibody (66911-1-Ig), anti-tubulin monoclonal antibody (66031-1-Ig), and DYKDDDDK tag polyclonal antibody (binds to FLAG Tag Epitope) (20543-1-AP) were purchased from Proteintech Group. β-Actin rabbit mAb (AC026), mouse anti HA-Tag mAb (AE008), rabbit anti HA-Tag pAb (AE036), and Myc-Tag rabbit mAb (AE070) were purchased from ABclonal Technology. Glyceraldehyde-3-phosphate dehydrogenase (GAPDH) mouse mAb (KM9002) was purchased from Sungene Biotech. EV-D68 VP3 polyclonal antibody (GTX132315) was purchased from GeneTex. The secondary antibodies were goat anti-rabbit horseradish peroxidase (HRP)-conjugated IgG (H + L) antibody (LK2001; Sungene Biotech) and HRP-conjugated anti-mouse IgG antibody (SA00001-1; Proteintech). The enhanced chemiluminescence reagent was obtained from Thermo Fisher Scientific.

Reactive Oxygen Species Assay Kit (CA1410), Mitochondrial Membrane Potential Kit (JC-10 Assay) (CA1310), 4′,6-diamidino-2-phenylindole (DAPI; H-1200), Hoechst 33342 stain solution (1 mg/mL) (C0031), and MitoTracker Red CMXRos (M9940) were acquired from Solarbio Life Science. TransScript II First-strand cDNA Synthesis SuperMix (AH301-02), FastPfu DNA polymerase (M21105), TransDetect Double-Luciferase Reporter Assay Kit (FR201-01), and TransStart Top Green qPCR SuperMix (AQ131-01) were purchased from TransGen (Beijing, China). Anti-Flag M2 Affinity Gel (A2220), Duolink *In Situ* Detection Reagents Red (DUO92002), Duolink *In Situ* PLA Probe Anti-Mouse PLUS (DUO92001), and Duolink *In Situ* PLA Probe Anti-Rabbit MINUS (DUO92006) were purchased from Sigma. Anti-HA Affinity Matrix (Product No. 11815016001) was purchased from Roche.

### Plasmids

NF-κB Luc reporter plasmids were purchased from Promega (Madison, WI, USA). Mammalian cell expression plasmids for MDA5 and TBK-1 with a FLAG-tag were kindly provided by XinYe and Shuai Chen (Institute of Microbiology, Chinese Academy of Sciences, Beijing, China). The FLAG-RIG-I plasmid was kindly provided by Jun Cui (Sun Yat-sen University, Guangzhou, China). The plasmid expressing MAVS and MAVS plasmid truncated mutants was generated by PCR amplification from HEK293T cDNA and cloned into the pCAGGS vector.

MAVS (forward, 5′-GTCTCATCATTTTGGCAAAGAATTCATGCCGTTTGCTGAAGACAAG-3′, and reverse, 5′-TTTGGCAGAGGGAAAAAGATCTCTACTTGTCATCGTCGTCCTTGTAATCGTGCAGACGCCGCCGGTACAG-3′), MAVS 142–540 (forward, 5′-ACGCGTCGACACCCCAGACCCACTGGAGCCACCG-3′, and reverse, 5′-TTTGGCAGAGGGAAAAAGATCTCTACTTGTCATCGTCGTCCTTGTAATCGTGCAGACGCCGCCGGTACAG-3′), MAVS 1–514 (forward, 5′-GTCTCATCATTTTGGCAAAGAATTCATGCCGTTTGCTGAAGACAAG-3′, and reverse, 5′-TTTGGCAGAGGGAAAAAGATCTCTACTTGTCATCGTCGTCCTTGTAATCCCCAGGTGAGGGCCTGTGGCA), and MAVS 1–455 (forward, 5′-GTCTCATCATTTTGGCAAAGAATTCATGCCGTTTGCTGAAGACAAG-3′, and reverse, 5′-AGATCTCTACTTGTCATCGTCGTCCTTGTAATCCCCCATGCCCAAGGAGGTGCTG-3′).

### RNA extraction and RT-qPCR

Total cellular RNA was extracted by TRIzol (Qiagen, Germany) extraction. Using TransScript II First-strand cDNA Synthesis SuperMix to reverse transcription of RNA to cDNA with oligo(dT) primers. RT-qPCR assay was performed using TransStart Top Green qPCR SuperMix, and mRNA levels were normalized to GAPDH mRNA. Results were analyzed as fold change using the 2^−ΔΔCt^ method.

Primers were used for EV-D68 VP1 mRNA (forward, 5′-GGCAGCCTATCAGGTGGAGAG-3′, and reverse, 5′-GAGTTTGTATGGCTTCTTCTGGT-3), IL-6 mRNA (forward, 5′-GGCACTGGCAGAAAACAACC-3′, and reverse, 5′-GCAAGTCTCCTCATTGAATCC-3), IL-10 mRNA (forward, 5′-AGAACCAAGACCCAGACATC-3′, and reverse, 5′-CATTCTTCACCTGCTCCAC-3), TNF-α mRNA (forward, 5′-CTCCTCACCCACACCATCAGCCGCA-3′, and reverse, 5′-ATAGATGGGCTCATACCAGGGCTTG-3), and GAPDH mRNA (forward, 5′-GAAGGTGAAGGTCGGAGTC-3′, and reverse, 5′-GAAGATGGTGATGGGATTTC-3).

### Coimmunoprecipitation and immunoblotting

HEK293T cells were transfected with the required plasmids. After 48 h, the cells were harvested and lysed with lysis buffer. The lysates were placed at 4℃ for 30 min and transferred 1/10 as input lysates to detect the total cell expression. The remaining lysates were incubated with Anti-Flag M2 Affinity Gel or Anti-HA Affinity Matrix at 4℃ for 6 h. The beads were washed six times with 1 mL of cold PBS and eluted with a glycine elution buffer to get immunoprecipitates. The samples in the SDS loading buffer were boiled and subjected to western blotting to detect protein expression. Western blotting was performed according to the conventional method as described previously (58).

### Luciferase reporter assays

NF-κB Luc plasmids, pRL-SV40, and experimental plasmid were co-transfected into HEK293T cells in 24-well plates. Cells were infected with SeV [20 hemagglutination assay units (HA)/mL] for 16 h prior to dual-luciferase reporter assay. All the operation steps were conducted strictly according to the instructions of the TransDetect Double-Luciferase Reporter Assay Kit.

### Immunofluorescence assay

RD/HeLa cells in 48-well plates plated with cell-climbing slices were transfected with plasmid or infected with EV-D68 and fixed with 4% paraformaldehyde for 15 min after 24 h. Cells were permeabilized with 0.5% Triton X-100 solution for 10 min and blocked with 1% bovine serum albumin (BSA) for 20 min at 37°C, and the primary antibody diluted in 1% BSA for 24 h was then incubated. Cells were incubated with fluorescent secondary antibodies for 40 min. Nuclei were counterstained with DAPI for 10 min. The cell climbing slices were mounted with 50% glycerol and observed under a confocal microscope.

### Proximity ligation assay

The interaction between EV-D68 VP3 and MAVS was tested using the Duolink *In Situ* Detection Reagents Red. HeLa cells in 48-well plates plated with cell-climbing slices were infected with EV-D68 or not for 24 h and fixed with 4% paraformaldehyde for 15 min. Cells were permeabilized with 0.5% Triton X-100 solution for 10 min and blocked with 1% BSA for 20 min at 37°C and incubated with anti-VP3 and anti-MAVS antibodies diluted in 1% BSA. Then, all the cells were incubated with anti-mouse minus and anti-rabbit plus PLA reagents, followed by ligation and amplification mix. Nuclei were counterstained with DAPI for 10 min. The cell climbing slices were mounted with 50% glycerol and observed under a confocal microscope.

### Reactive oxygen species assay

HeLa cells were seeded in 48-well plates plated with cell-climbing slices and infected with EV-D68 of different MOIs (MOI = 0.01, 0.05, 0.1). After 24 h, the samples were incubated with DCFH-DA probe (1:3000) and Hoechst (1:100) at 37°C for 30 min and observed under a confocal microscope.

### Mitochondrial membrane potential detection

RD cells were seeded in a 6-well plate and transfected or infected with EV-D68 at different MOIs (MOI = 0.01, 0.05, 0.1) as required. The JC-10 working solution was prepared according to the manufacturer’s instructions. Cells were harvested and resuspended in 500 µL of medium. Cells were incubated with 500 µL of JC-10 working solution at 37°C for 20 min. Cells were washed twice with JC-10 staining buffer (1×) and resuspended. The samples were analyzed using a flow cytometer, and data were processed with CytExpert software.

### Statistical analysis

Data were analyzed with GraphPad Prism and expressed as mean and standard error. Statistical significance was determined using two-tailed Student’s unpaired *t*-test or one-way analysis of variance (n.s. not significant; *P < 0.05; **P < 0.01; ***P < 0.001; ****P < 0.0001).

## Data Availability

All of the data are fully available without any restriction.

## SUPPLEMENTAL MATERIAL

Supplemental figures jvi.00163-25-s0001.pdf Fig. S1 to S4.

ASM does not own the copyrights to Supplemental Material that may be linked to, or accessed through, an article. The authors have granted ASM a non-exclusive, world-wide license to publish the Supplemental Material files. Please contact the corresponding author directly for reuse.
