# Supplementary material for: The structural protein VP3 of enterovirus D68 interacts with MAVS to inhibit the NF-κB signaling pathway
Source: J Virol. 2025 Mar 5;99(4):e00163-25. doi: 10.1128/jvi.00163-25 (PMC11998529; doi:10.1128/jvi.00163-25)
Supplement: Supplemental figures — Fig. S1 to S4. [file jvi.00163-25-s0001.pdf]

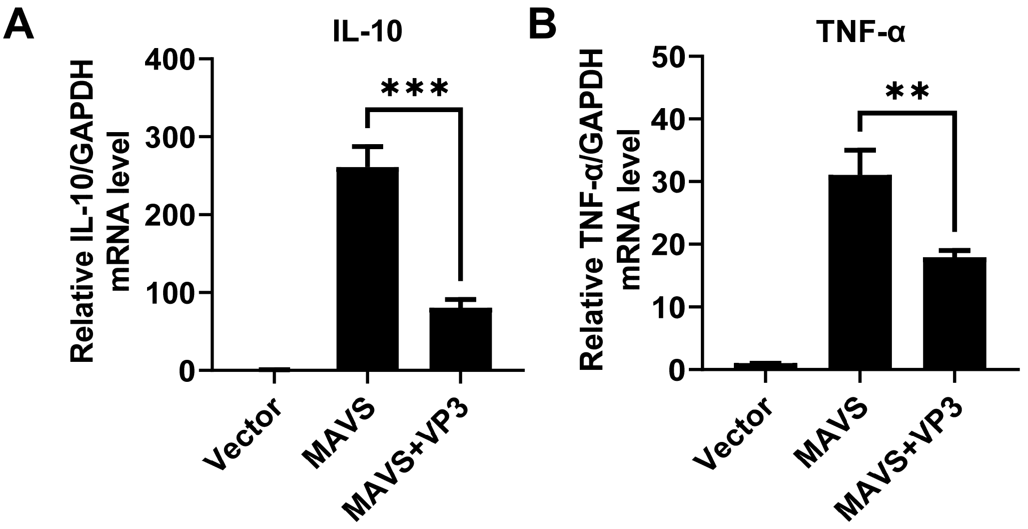

Fig S1. VP3 inhibits the transcriptional levels of downstream genes in the NF-κB signaling pathway. (A, B) RD cells were co-transfected with Vector, MAVS or MAVS+VP3. After 36h, Cells were collected for extracting total RNA and the expression of (A) IL-10 and (B) TNF-α mRNA was determined by RT-qPCR assay. Results are expressed as mRNA levels relative to GAPDH mRNA levels.

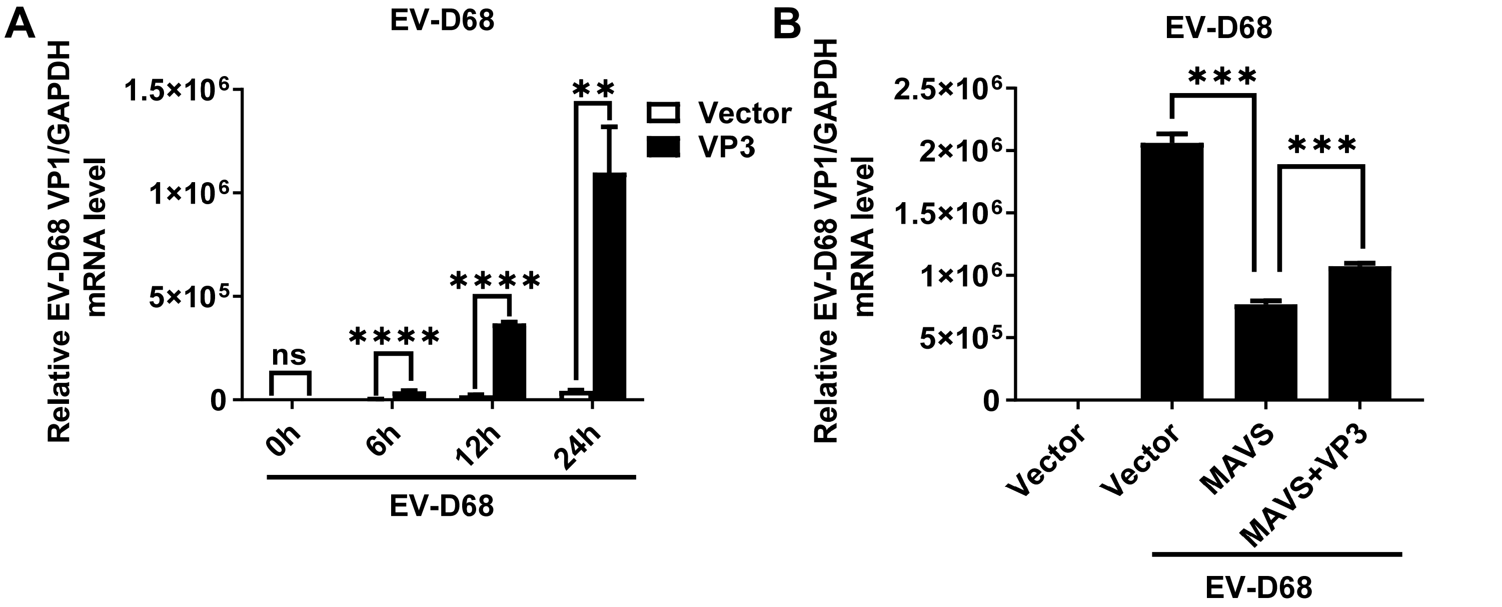

FigS2. EV-D68 VP3 overexpression promotes virus replication. (A) RT-qPCR was used to detect the effect of VP3 on EV-D68 virus replication at different time (0 h, 6 h, 12 h, 24 h) during the EV-D68 infected HEK293T cells process. (B) RD cells were transfected with Vector, MAVS or MAVS+VP3. After 24 h, EV-D68 (MOI = 0.1) infected the cells, and the relative level of EV-D68 mRNA was detected by RT-qPCR. The RT-qPCR results are shown as changes in mRNA levels in the infected group relative to the uninfected group, and normalized to GAPDH.

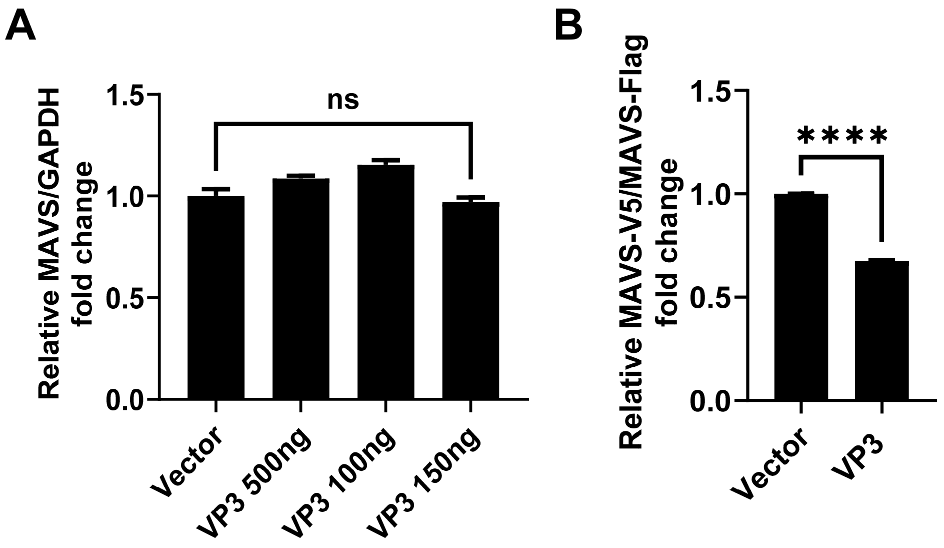

Fig S3. Gray-scale analysis of partial Western blot images. (A) The gray level of MAVS and GAPDH from Fig 2A were quantified by ImageJ. (B) The gray level of MAVS-Flag, MAVS-V5 and GAPDH from Fig 3G were quantified by ImageJ.

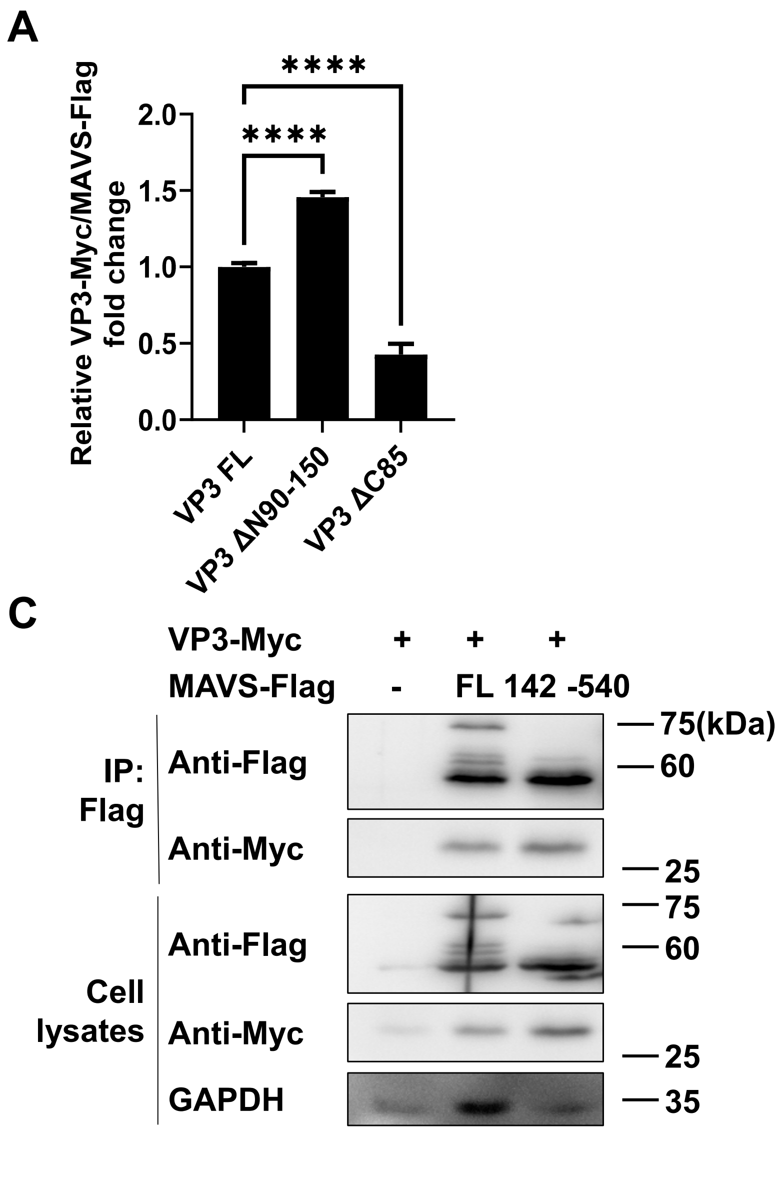

Fig S4. The interaction site of EV-D68 VP3 with MAVS. (A) The gray level of MAVS-Flag, VP3-Myc from Fig 5C were quantified by ImageJ. (B) The gray level of MAVS-Flag, VP3-Myc from Fig 5E were quantified by ImageJ. (C) HEK293T cells were co-transfected with 3 μg of VP3-Myc plasmid and 3 μg of Vector, MAVS FL, MAVS 142-540. Cells were harvested after 48 h for co-immunoprecipitation experiments. (D) The gray level of MAVS-Flag, VP3-Myc from Fig S4C were quantified by ImageJ.
